# The role of information search in seeking alternative treatment for back pain: a qualitative analysis

**DOI:** 10.1186/2045-709X-22-16

**Published:** 2014-04-11

**Authors:** Hoda McClymont, Jeff Gow, Chad Perry

**Affiliations:** 1 School of Management and Enterprise, University of Southern Queensland 4350 Toowoomba, QLD, Australia; 2 School of Commerce, University of Southern Queensland, Toowoomba, Australia; 3 Research Associate, Health Economics and HIV/AIDS Research Division (HEARD), University of KwaZulu-Natal, Westville Campus, Durban, South Africa

**Keywords:** Back pain, Information search, Search effort, Complementary and alternative treatments, Australia

## Abstract

**Background:**

Health consumers have moved away from a reliance on medical practitioner advice to more independent decision processes and so their information search processes have subsequently widened. This study examined how persons with back pain searched for alternative treatment types and service providers. That is, what information do they seek and how; what sources do they use and why; and by what means do they search for it?

**Methods:**

12 persons with back pain were interviewed. The method used was convergent interviewing. This involved a series of semi-structured questions to obtain open-ended answers. The interviewer analysed the responses and refined the questions after each interview, to converge on the dominant factors influencing decisions about treatment patterns.

**Results:**

Persons with back pain mainly search their memories and use word of mouth (their doctor and friends) for information about potential treatments and service providers. Their search is generally limited due to personal, provider-related and information-supply reasons. However, they did want in-depth information about the alternative treatments and providers in an attempt to establish apriori their efficacy in treating their specific back problems. They searched different sources depending on the type of information they required.

**Conclusions:**

The findings differ from previous studies about the types of information health consumers require when searching for information about alternative or mainstream healthcare services. The results have identified for the first time that limited information availability was only one of three categories of reasons identified about why persons with back pain do not search for more information particularly from external non-personal sources.

## Background

Health consumers are moving away from a heavy reliance on medical practitioner advice to more independent decision processes [1,2]. In countries such as Australia, the United Kingdom and the United States shared decision making and patient self-management are key components of health policy [3]. There are five steps to the consumer health decision making process: need recognition; information search; evaluation of alternatives; purchase decision and post-purchase behavior [4]. In this paper a qualitative examination of the second step, information search, in managing chronic back pain is undertaken.

Information search is particularly relevant to consumers with chronic diseases where doctors cannot recommend one best treatment that will solve the problem [2,5]. One such chronic condition is back pain caused by musculoskeletal disease. Traditionally, information about solutions to back pain and the available treatments has been provided by mainstream health providers such as physiotherapists and, in more serious cases, by surgeons. However, more recent trends indicate that an increasing number of persons with back pain are turning to alternative or complementary treatments such as chiropractic treatments, homeopathy, naturopathy, Bowen therapy, kinesiology and reflexology [5]. This change in treatment direction combined with the multitude of treatment solutions necessitates factual and effective information in order to guide consumer’s choices.

This area of research is particularly important because back pain is a significant problem affecting people globally [6]. In industrialised countries, more than 80 per cent of people will experience back pain sometime in their life and up to half of the workforce will suffer at least one episode of back pain each year resulting in work absences, lower productivity and increased costs to businesses [6]. For example, half of all working Americans experience back pain each year and it is one of the most common reasons for missing work [7]. In the United Kingdom, there are approximately three million persons with back pain and of the 131 million working days lost to sickness, 34.4 million are due to musculoskeletal conditions such as back pain, neck pain and limb problems [8]. In turn, musculoskeletal disorders are the leading cause of disability in Australia; they accounted for more disability than any other medical condition with 14% (2.8 million people) experiencing back pain and disc disorders [9].

Despite this significance of back pain problems and a trend towards alternative information sources and treatment options, little is known about the information search processes used by persons with back pain to select appropriate treatment types and service providers in particular. This gap is important because persons with back pain search behavior are the *start* of any treatment chain. Other research has addressed search behavior by patients and/or patients’ families searching for information about areas like hospitals, physicians and healers [10-12], aged care homes [11], mental health services [13] and cancer [14,15]. Recent research has covered search behavior on the internet for health related issues that did not include back pain [16,17]. But persons with back pain overall information search behavior has not been researched [18] and so requires some qualitative investigation to uncover.

This study aims to extend previous research on information search in healthcare by using the convergent interviewing methodology to investigate how persons with back pain apply three essential elements of the search process to acquire information about treatment types and service providers: what sources do they use and why; what information content do they seek and how; and how do they search for it?

The next section briefly reviews literature on information search in the health care context. Then data collection and analysis methods of convergent interviewing used in this research are described. Analysis of the research data to develop schemas of information search behaviour by persons with back pain, and their implications, follow.

### Prior theory - information search

This research extends prior theory to back pain situations and so that theory needs to be established first. Accessing the right information efficiently reduces uncertainty and anxiety [2] and guides the decision making of health consumers. To undertake this information search, a typical consumer has to decide on three key issues that became the three research questions about back pain that drove the data collection and analysis phases of this research: where to search for it [19], what information content to look for [20], and how much search effort to expend when looking for information [21].

### Information sources

Firstly, consider sources of information. The literature on information search identifies two main sources: internal and external sources. Internal search comprises searching one’s memory to access information about solutions to an existing problem [19,22,23]. This information is basically stored knowledge and experiences gathered over time [24]. Thus prior knowledge is a key to undertaking any effective search for it provides the basis for evaluating new information in later, external search. Furthermore, prior knowledge has been shown to influence both the amount and type of information sought. However, this influence appears to be context specific [25] with certain contexts leading to more information search with greater knowledge while others lead to less information search with greater knowledge [26]. Despite its contextual nature, no studies were found on the use of internal information search within a health care context. Thus, the question arises about how much consumers with back pain rely on their memories (internal sources) for treatment type and service provider information and how does this internal search impact the amount and type of external information search?

In turn, if internal information is deficient, external information sources are usually used. External information search constitutes seeking information from the outside environment [24]. In contrast to the scant research about internal sources, some research findings are available about external information sources used to make health treatment and service provider selections [10,27-29]. For example, [30] suggested that information about mainstream health care services is effectively distributed through external sources such as pamphlets, health professionals and formal educational programs. However, other studies indicate that information search by patients does not rely on these sources and instead uses more “personal” external sources; for example, information about general health matters could be sought from family members, friends, work colleagues and doctors [1,31]. For back pain, studies show booklets and physical-related cues might be effective but other sources like video are not [32,33]. Other external sources about back pain are online [34,35]. But are these *all* the sources for back pain information? This was the first research question.

### Information content

The second issue raised in the information search literature is the nature of the content of the information patients look for or indeed avoid looking for [12,36]. The information content sought by health consumers is context specific [20]. For example, [37] found that women going through menopause searched for information to work out which symptoms were ‘normal’ for their age group; which symptoms they experienced were related to menopause; to get information to prepare for their visit to the doctor, and to later confirm the doctor’s diagnosis and expand their knowledge of issues raised by the doctor. As another example, parents of children often have concerns and require specific information to combat misconceptions, and address their doubts about the safety/efficacy of treatments [38]. A final example relates to female cancer patients’ whereby women search for information topics relating to therapies available, how to manage a recurrence of cancer if a treatment stops working, types of surgery that can be undertaken for the cancer and pain issues [39]. What context-specific information do persons with back pain seek? This was the second research question.

### Search effort

The third element of information search behavior is the search effort expended by consumers. While the search effort construct [40,41] is well recognized and accepted in the marketing literature, there does not appear to be an accepted way to measure it. For example [42], measured this construct using two items: the amount of time and the amount of effort used to locate information [43] also measured search effort for home shoppers using the single item of the number of advertisements referred to in catalogues [21] defined search effort based on the number of sources consulted and the amount of effort required to gather and comprehend information. In health care research [44], measured internet search effort based on a single scale of measuring the extent of agreement/disagreement with the statement: “it took a lot of effort to get the information you needed”. Other health care research investigating information search behaviours appear to ignore this issue totally [45]. In brief, search effort has been measured using time, number of sources, effort level or a combination thereof. But what defines search effort by persons with back pain? This was the third and final research question.

## Methods

Qualitative research methods are appropriate for the investigation of how and/or why a social phenomenon occurs, and are therefore appropriate for this study’s research problems [46,47]. That is, “the aim of qualitative research is to develop concepts that can help us understand social phenomena in natural settings, giving emphasis on the meanings, experiences and views of the participants” [48], p.11. The qualitative method of convergent interviewing was selected. It involved a series of long, initially rather unstructured interviews [49-51]. The interviewer analysed the data and refined the interview questions after each interview, to converge on the emerging issues in the topic area. In brief, flexible exploration of this complex and sensitive topic was possible through the convergent interviewing technique.

This convergent interviewing methodology is *justified* for this research in four ways [50]. Firstly, it converges quickly on important issues. The required number of interviewees in convergent interviewing should be less than traditional interviewing’s because any points of convergence or divergence among interviewees are examined after each interview to develop the questions and probes for the next interview; that is, the methodology is a thorough one. Moreover, this efficiency is important when interviewees are time-limited, like many of the busy interviewees in this research project. Secondly, convergent interviewing has a mechanism for knowing when to stop collecting data – the “stability” described below. Finally, it sets a sound stage for further research methodologies such as case research.

However, there are limitations related to convergent interviewing as a research methodology. Firstly, convergent interviews are time consuming and require many consecutive interviews with many different people [46]. But the analysis after each interview allowed a convergence on key issues, and the use of the prior theory permitted a focus on important issues. Secondly, there is a risk of bias because the researcher, as the interviewer, is a participant in the data collection process. This risk was mitigated by the researcher’s understanding and experience of the methodology and her use of appropriate interview techniques [52]. Moreover, the methodology’s analysis after each interview reduces bias that may exist in traditional interviewing’s one-off data analysis process that starts after the final interview. Indeed, the methodology could perhaps be called ‘convergent and divergent interviewing’ because explanations for any differences of opinion are probed for in each interview. Next, convergent interviewing is an exploratory technique and therefore should be used in conjunction with later research, and this limitation is acknowledged in the ‘further research’ section below. Finally the lack of interviewee validation could result in misinterpretation of the results by the researcher, but asking interviewees to decide the validity of the social-scientific rendering of interpretations of the interviews would be unreasonable because of their lack of discipline-specific knowledge [53].

Interviews were conducted with adult persons with back pain who had sought either one or more types of treatment for their problem. Study participants were recruited in Australia through notices in physiotherapy practices, government offices and one university. The twelve interviewees were purposively selected to ensure gender, age and educational balance in the sample, as shown in Table 1.

**Table 1:** Sample characteristics of convergent interview interviewees (n = 12)

	**Frequency**		**Frequency**
**Gender**		**Highest education level**	
Females	5	Secondary	5
Males	7	Post secondary	2
		Tertiary	5
**Age**			
Less than 25 years		**Occupation**	
25 – less than 40 years	2	Professional	6
40 – less than 60 years	3	Semi-professional	2
	7	Trades	1
		Student	2
		Disability pension	1

A key question is, “How many interviews should have been conducted?” The answer to this question about convergent interviewing revolves around the concept of “stabilty” or “saturation” when no new information or patterns in the data emerge from the interviews [54]. This stabilty depends on the skill of the interviewer, the type of research problem, the cases themselves, and the depth of data analysis [55], p.245 summarises this difficulty of deciding how many interviews are needed: “The validity, meaningfulness and insights generated from qualitative inquiry have more to do with the information-richness of the cases selected and the observational/analytical capabilities of the researcher than with sample size.” The interviewer was an experienced qualitative researcher who had previous interviewing experience and who had a working knowledge of the subject matter.

Estimates of the required number of interviews vary. That minimum can be about six [54,55], 10 [56] or 15 [57]. The maximum can be about 50 [56,58]. Stability is often reached after only five or so convergent interviews [50]. The final convergent interview occurs when interview data analysis of that interview shows there is stability through a consistent pattern of agreements and disagreements in the last two interviews [46,50,51]. In this study, this stability appeared after the eleventh interview. To ensure that stability had been reached, an additional interview was conducted but no new information emerged.

At each interview, the researcher clarified a number of administrative issues including ethical clearance and whether the interview could be taped. The opening question was broad [46] and then detail-oriented probes, elaboration probes, and clarification probes were used within the interview [59]. Incidentally, “back pain” is defined in this report in general terms: “a pain in the lumbar, lumbosacral, or cervical regions of the back, varying in sharpness and intensity. Causes may include muscle strain or pressure on the root of a nerve” [60]. Each interview ran for approximately 60 minutes and was taped and then transcribed before analysis. Each interview was transcribed within a day of the interview and the transcript was triangulated with the notes taken by the researcher during the interview. The interviewer undertook preliminary analysis of each of the interview transcripts and accessed literature about issues that emerged as a result of the interview in order to better understand the subject matter. These steps allowed the researcher to modify the interviewer’s guide as necessary (in line with the convergent interviewing methodology) to obtain more detailed and relevant information in the following interviews.

Data reduction was undertaken to condense data to assist in the development of a conceptual framework. Thus, transcripts were coded into themes and sub-themes according to the issues identified during the literature review. The three themes were information sources, information content and search effort although each of these were further divided into sub-themes such as internal information sources, external personal sources and external non-personal sources under the information sources theme. Where new and contrary information was found in the data, these were coded to new categories and discussed later. Next, the data from the various coded themes and sub-themes were linked together to find regularities and make sense of the data [61]. These regularities were tallied in a table next to each theme or sub-themes and the interviewee number and quote recorded alongside them.

In order to validate findings and their interpretations, one researcher undertook the analysis and discussed the findings with the other researchers. Next, the analysis was put aside for some time and then returned to by the researcher and re-analysed. This action resulted in some minor errors in the initial interpretations. These were rectified by revisiting the interview transcripts to clarify what the interviewees had said and assessing the logic with which ideas were categorized into a theme or sub-theme based on prior literature and data reduction techniques.

Ethical approval was obtained from the University of Southern Queensland’s Human Research Ethics Committee (Ethical Approval number: H05REA495).

## Results and discussion

### Research question 1: what sources of information do persons with back pain use, and why?

Persons with back pain use a variety of internal, external personal (subjective) and external non-personal (objective) information sources during decision making, as summarized in Table 2, where the relative importance of each item is estimated.

**Table 2:** Information sources used to select treatment types/service providers

**Information sources**	**Interviewees**	**No. of interviewees using each source**	**Rank of frequency of sources used**
**Internal source –** memory of past knowledge and experiences	1, 2, 3, 4, 5, 7, 8, 9, 10, 11, 12	6	2
**External personal sources**			
Word of mouth – friends	1, 2, 4, 5, 7, 9, 11	7	1
Doctor/other therapists’ referrals	1, 4, 5, 7, 9, 11	6	2
Word of mouth – family	1, 5, 8, 9	4	3
Word of mouth – work colleagues	1, 2, 6, 12	4	3
Speaking with a provider prior to appointment	2, 10	2	5
Word of mouth - other (for example, teacher, receptionist at my doctor’s practice)	3, 10	2	5
**External non-personal sources**			
Yellow/white pages	1, 2, 3, 8	4	3
Promotional sign at provider’s office	2, 3, 11	3	4
Internet	5, 7	2	5
Books	5	1	6
Radio talk back show	7	1	6

One of the two most important sources of information is *internal*. Almost all interviewees (11 of 12 interviewees) stated using internal sources in one or more of their decision making situations for back pain treatment selection This frequent use of internal information sources (including past knowledge and/or personal experiences) reflects other consumer research about services that require experience to evaluate [4] and indicates that persons with back pain tend to rely on their own memories. But how comprehensive and accurate is their internal information?

In order to ascertain how accurate and comprehensive interviewee’s knowledge and experiences were, they were asked to explain why they chose the treatment type that they had. The majority (66%, or interviewees 2, 3, 4, 5, 7, 8, 9, 12) of interviewees explained that they had made their choices based on their perceptions of their back problem, their perception of what each treatment type could offer and thus which would be most suitable for their problem. For example, interviewee 9 said:

‘I thought my muscles were tight from sitting at the computer every day at work so the masseuse would relax my muscles. I didn’t think I had a medical problem to be sorted out by a chiropractor or physiotherapist’.

#### *Misconceptions and biases about chiropractors and physiotherapist*s

An interesting finding was that most interviewees had misconceptions of the nature of their problem and workings of chiropractic and physiotherapy treatments even though these treatments were the ones most used by interviewees. Some misconceptions were:

•That they perceived their bones were ‘out’ of their spine’ and that they needed to be ‘put back in’

•That physiotherapists have nothing to do with treating pain and muscle spasm:

•That physiotherapists and chiropractors only deal with injuries and rehabilitation

•That chiropractors are only helpful for non-specific back pain

•That physiotherapists are better than chiropractors or vice versa

*Chiropractors manipulate the bones and put them in so they loosen the muscles but they don’t retighten them. Physiotherapists don’t actually put the bones in but they make the muscles work so that the bones go in by themselves. I know this from my suppositions mainly and from talking to physiotherapists* (Interviewee 3).

*But, I knew chiropractors put bones back into place and so in my first accident when I popped a vertebra out I knew a chiropractor would put the bones back into place … I picked [Bowen Therapy] because I thought that chiropractors put people’s bones into place but I also knew that muscles keep your bones in place and so there is no point getting your bones put into place if they (your muscles) are not working right. So I wanted to go to Bowen therapy* (Interviewee 5).

*I chose a chiropractor because I knew it was something structural – something was ‘out’ and it had to be put back in. And my perception is the physiotherapists work more with muscles and give massage and exercise things. But I felt like something had gone out so I just felt like I needed to have it pushed back in* (Interviewee 9).

*When people think of physiotherapists, they don’t think of them as people who could help heal muscle spasm and pain. And people think of chiropractors as aligning the back which has little to do with muscle pain and spasm* (Interviewee 2).

*I liked the notion that chiropractors could deal with the manipulation ….like the operation of a chiropractic move. Like, they are realigning what you have. So if your back is causing you grief due to the tension that you may be carrying around then they can realign the neck and fix your headache. Whereas other treatments, I am not sure how they work but I don’t like the idea of taking a tablet for a headache. I would rather deal with the cause. I always associate physiotherapists with sports injury and rehabilitation (for example, people who have their arm in a cast and they build the muscle up.’* (Interviewee 10).

*Chiropractors are good for general back pain which I initially had. The treatments allowed me to wake up in the mornings without feeling stiff. They straightened my back which was crooked and I will go back for my general back pain to them but not for the* spondylolisthesis*. I try to stretch my treatments to 4–5 weeks now* (Interviewee 7).

*‘I thought of seeing a chiropractor but what I had learnt about physiotherapists and chiropractors was the physiotherapists were better’* (Interviewee 4).

#### Knowledge and experience void relating to alternative treatments

Unlike the multitude of opinions provided about physiotherapy and chiropractic care, some interviewees appeared not to know as much or express strong opinions about other treatment types (interviewees 5, 6, 7, 10, 11). Therefore, they relied mainly on their emotions and memories of vicarious learning and recommendations made by others to select a treatment type. For example, interviewee 7 chose an acupuncturist because of desperation rather than because of his knowledge of the treatment: ‘*I chose this type of treatment out of desperation and the lack of success with physiotherapists and chiropractors. Also, I was curious about this type of treatment and wondered what benefits it could have.’* Interviewee 10 chose chiropractors because she did not like needles despite the fact that acupuncture needles do not function in the same way that injections do: *‘ I always associate physiotherapists with sports injury and rehabilitation (for example, people who have their arm in a cast and they build the muscle up. And, I don’t like acupuncture because I don’t like needles’*. Interviewee 5 said: ‘*I found out about Reiki from mum and her books’. Respondent 1 used memories of vicarious learning to select a treatment type: ‘I didn’t really know anything about physiotherapists and chiropractors other than they help with pain. But I knew that my sister had had luck with a chiropractor and so I just chose to go to them’* (Interviewee 1).

However, once interviewees had had back problems for some time and had experienced a treatment, they used various criteria to decide on the treatment type and provider. Therefore, their criteria for selection included their past knowledge as well as their present experience with which to construct their decision.

### Biased attitudes towards non-mainstream treatments

Some interviewees revealed biases against non-mainstream treatments. For example, interviewee 11 was biased against non-mainstream treatments because of his rural upbringing: *‘The others (acupuncture, osteopath or any treatment outside of physiotherapy and chiropractors) are voodoo. … well I come from a rural background and family members and people I associate with said to go to a physiotherapist. You wouldn’t go to a chiropractor because they could injure you, crack your bones. As far as any alternative things like acupuncture – they are extreme’*.

Similarly, interviewee 4 showed bias toward alternative treatments like chiropractic care based on his experience:

I thought of seeing a chiropractor but what I had learnt about physiotherapists and chiropractors, was the physiotherapists were better. I knew a chiropractor as a friend when I told him my symptoms, he said that I am better off with a physiotherapist who works with musculoskeletal issues rather than spinal issues (which is what a chiropractor does).

#### External sources

Persons with back pain also rely on *external* personal sources to provide credence about the treatment and service they seek. Two external personal sources are word of mouth by friends, and referrals by doctors and other therapists. Other external personal sources used include word of mouth by family and work colleagues, speaking with a provider prior to booking an appointment, and speaking with others such as teachers and receptionists.

External non-personal sources are not used as much as external personal sources, because they do not have the credence of personal sources [4], as discussed below. These external non-personal sources include the telephone directory, advertising signage outside the provider’s office, the internet, books and talk-back shows.

No interviewee relied solely on an internal source, even if they have past experience and knowledge to fall back on – all those using an internal source also used an external personal source, and some of them used an external non-personal source as well. Clearly, for back pain services, credence is required as well as experience [4].

#### Effectiveness of external personal sources

One question that remains unanswered in the literature is how effective are external personal sources in achieving positive treatment outcomes? To investigate this issue, a summary of all incidents in which interviewees outlined information sources used to select a treatment type and subsequent treatment outcomes was developed (refer Table 3).

**Table 3:** External personal sources used to select treatment types/service providers

**Interviewee**	**External source**	**1**^**st **^**ever treatment**	**2**^**nd **^**treatment**	**3**^**rd **^**treatment**	**Treatment outcome**
1	WOM – family		Pain subsided from chiropractor		Positive
1	WOM - friend			Pain subsided from physiotherapy	Positive
2	WOM - colleague		Pain subsided from masseuse		Positive
3	WOM – teacher		Paid subsided from chiropractor		Positive
4	GP - referral	Pain relief but problem progressively worsened with physiotherapy			Negative
4	GP - referral		Some pain relief but got problem got progressively worse with physiotherapy		Negative
4	GP - referral			Some pain relief but got problem got progressively worse with physiotherapy	Negative
5	WOM – friends	Inadequate length of pain relief from chiropractor			Negative
5	WOM – family		No pain relief with colour therapy		Negative
5	WOM – family			Inadequate length of pain relief	Negative
6	WOM - colleague	Unhappy with chiropractor’s behaviour			Negative
6	WOM – friends		Inadequate length of pain relief from acupuncture		Somewhat positive
6	WOM – friends			Inadequate length of pain relief from acupuncture	Somewhat positive
7	GP and WOM – friends	Not totally pain free with physiotherapy			Somewhat positive
7	WOM – friends		In adequate relief of symptoms with chiropractor		Negative
7	WOM - friend			No pain relief from acupuncture	Negative
8	WOM - mum	Adequate pain relief with chiropractor			Positive
8	WOM and marketing sources		Adequate pain relief with chiropractor		Positive
9	WOM friends/others	Adequate pain relief with chiropractor			Positive
10	Chiropractor’s referral		Adequate pain relief		Positive
10	Chiropractor’s referral			Adequate pain relief	Positive
11	WOM –friends	Inadequate pain relief			Negative
11	GP referral		Adequate pain relief		Positive
11	WOM – friend and family			Inadequate pain relief	Negative
12	WOM - friend	Adequate pain relief			Positive
12	WOM - friend		Adequate pain relief		Positive
All	Total treatment outcomes				**26**
All	Total ‘somewhat positives’ outcomes*				**3 (11.5%)**
All	Total ’positive’ outcomes				**12 (46.2%)**
All	Total negative outcomes				**11 (42.3%)**

*Note: somewhat positive outcomes frequency was excluded from the final calculation because of the outcomes were not satisfactory enough for the patient to remain loyal to the practitioner.

Table 3 summarises 26 incidents where interviewees had selected a treatment provider based on word of mouth. Of the 26 incidents, only 12 (46.2%) had positive outcomes. A positive outcome is defined as adequate relief of symptoms for an acceptable period of time as judged by the respondent. The other 14 incidents were 11 (42.3%) who had no symptom relief (a negative outcome) and another 3 (11.5%) who had some symptom relief but were not fully satisfied (a somewhat positive outcome). In brief, we propose that although word of mouth is heavily relied on for decisions about which treatment type and provider to use, it might not be the most effective source to use. Further research is warranted to establish the validity of this finding and to identify any other factors which may be contributing to this outcome.

In brief, the findings of the internal and external information sources indicated that persons with back pain have and obtain little if any accurate information about the nature of their problem and what different treatments can and should be used for. As a result, interviewees’ final choice of treatment type is based more on trial and error. This finding is supported by a cancer study in which its noted that many patients do not know how to access credible information and make informed decisions about cancer [62].

One poorly resolved issue in the literature relates to the reasons for the limited use of some information sources and this research makes a contribution about this issue. In particular, why are not more external non-personal sources used? Three categories of reasons for *not* searching more extensively for external information about treatment types or service providers emerged in the interviews. First, many interviewees provided *personal* reasons for not searching for information such as:

•A lack of interest as noted by interviewee 11:

•Not wanting to self- diagnose:

•Being able to self-diagnose as stated by interviewee 9: ‘No I didn’t look for any other information because being a nurse, you self-diagnose a lot.’

I don’t look for much information about what my problems are or treatments because usually you are so busy and flat out that you, sort of, are very reactive in everything you do….

*Nowadays I suppose you could look on the internet but I don’t reckon I would do that probably because I would rather be led by an expert rather than have a look at a whole lot of stuff and start deciding…* (Interviewee 12).

The second category of reasons for limiting search of external non-personal sources can be labeled provider-related. Two reasons were offered:

•Loyalty to the treatment provider. For example, interviewee 3 said that her loyalty stopped her from searching for information: “He [the physiotherapist] was the one I went to 25 years ago after my high jump accident, I had success with him and I was … loyal.”

•The provider provided sufficient information about their treatment relative to other providers.

*I didn’t look for any other information because on my first consultation with the chiropractor, they took me through what chiropractic care is and how it differs from a doctor, physiotherapist or acupuncturist. So I felt they gave me enough information* (Interviewee 10).

Finally*, information-supply reasons* for limited use of external sources included reasons such as patients not knowing what information to look for, where to look for information, or the information not being readily available. For example, interviewees 7 and 12 both believed that information was not readily available. Interviewee 7, who suffers from spondylolisthesis, a rare spinal pressure produced by the forward dislocation of one vertebra over the one beneath it, said that he had only looked for information about this problem on the Internet because it is a very specific condition and information could not be found through general sources such as general interest magazines. Interviewee 2 also found it difficult to find information readily and said:

It was difficult to find information about back pain. The Yellow Pages [telephone directory] just had names and contact details of physiotherapists and chiropractors but they didn’t have a list of people who could assist with back pain.”

In turn, interviewee 1 did not know what information to look for or where to look for it: “*I didn’t look for information because I didn’t know what to look for or where to look for it … there wasn’t any real information in your face.”*

In brief, internal and only external *personal* sources of information are most important for persons with back pain. External non-personal information in not as used because of personal, provider or supply related reasons.

### Research question 2: what information content is sought by persons with back pain, and how?

Interviewees sought information about two items: their *treatment options*, and their choice of service providers within a selected treatment option. The content sought about treatment options included information about the types of back problems that exist and their symptoms; the types of treatment options that exist and the specific symptoms they address or benefits they have; how each treatment option works and its origins; the efficacy of each treatment option; and self-help tips.

In addition to information sought about treatment options, interviewees wanted information about *service providers* including:

1. Finding service providers within each treatment type and their area of expertise. For example, interviewee 6 said: *“I ended up talking to friends to find out … which ones [acupuncturists] were good to go to.”*

2. Service providers’ reputation. For example, interviewee 10 said:

*“I went and saw Dr X and he fixed my neck and I went out and my mum was talking to her [the receptionist] about my prescription and how Dr X realigned my neck and she said “I go to a really good chiropractor if you are looking for one” and then she suggested X Chiropractic*.”

3. Provider’s contact details. For example, interviewee 8 said: *“I found out about him through mum … Then I jumped on the Internet for the White Pages to find his number…”*

4. Proximity to provider. For example, interviewee 1 looked through the Yellow Pages to locate a chiropractor close to where she lived. In turn, interviewee 2 said that he was in pain and walked by the masseuse therapist’s shop and decided to go in for a massage. He made this decision because he was in city Y at the time that he experienced this pain and so could not wait to see his masseuse back home in city X.

5. How speedily an appointment could be obtained with the provider. For example, interviewee 11 said: *“And I needed someone that I could get into straight away…”*

6. Accessibility of the provider to discuss matters before making an appointment. For example, interviewee 2 searched for information about a provider by phoning a masseuse therapist, telling him about his problem and asking him what he could do about it.

### The relationship between information content and information source

**Figure 1:**
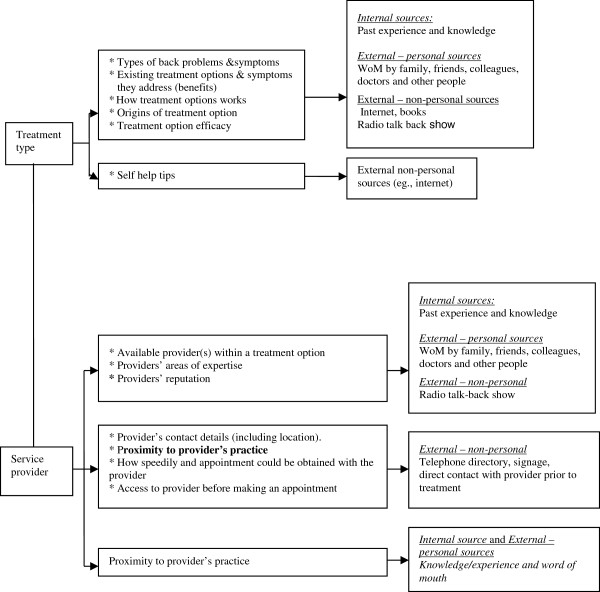
Mapping the relationship between information content and information sources.

Another issue, not highlighted in the extant literature, emerged from the interview data: there was a link between some information items sought about treatment options and about service providers, and the types of information sources used to locate such information. Figure 1 shows these linkages, with treatment options and service providers on the left and information sources on the right.

To begin, information about *treatment options* was sourced through internal sources (when it was available), and through several external personal and external non-personal sources, as shown in Figure 1. Next, consider the findings about the linkages about *service provider* information and information sources, as also summarized in Figure 1. These linkages had three patterns. First, information about the availability of providers practicing a treatment type and their reputation and expertise was sourced mainly through internal sources (such as past experience) and external personal sources including word of mouth from friends, family, colleagues, doctors and others. Second, very specific information about a service provider such as the contact details, location of practice, speed of obtaining an appointment and access to the provider before an appointment, was sourced mainly from external sources such as the telephone directory, signage outside the practice and through direct contact with the provider prior to treatment bookings. Third, information about the location of a service provider’s practice was also sourced through internal sources (that is, past experience and knowledge) and through external personal sources of word of mouth.

In brief, the context-specific items of information sought by persons with back pain were identified for the first time, and the linkages between their sources of information and type of information were identified for the first time for any health care patient.

### Research question 3: How do persons with back pain search for information?

The meaning of “search effort” emerged as the number of sources used and whether a patient deliberately or incidentally looked for information prior to making a decision. The term “deliberately” refers to situations where interviewees made a deliberate decision to look for information. By contrast, situations are categorized as “incidentally” when interviewees were exposed to certain information even though they were not deliberately searching for it. This distinction between deliberate and incidental search effort has not been identified before.

First, consider *deliberate* search through the examples provided by interviewees. Naturally, the decision was a deliberate one when the interviewee referred to their memory banks (internal information) to select a suitable treatment type and service provider (for example, interviewees 3, 8 and 9). Similarly, deliberate search was undertaken by interviewees who sought information externally through the Yellow/White Pages, the internet, books, doctors, speaking with service providers before the appointment and, in some cases, speaking with family members (for example, interviewees 1, 2, 4, 5 7, 8 and 10).

Interviewees who deliberately consulted family members about their back problems and sought assistance from them, were dependents living at home. Of the five interviewees (interviewees 1, 3, 5, 8 and 10) who did consult family members, four did so to obtain assistance. For example, the younger, dependent interviewees 3, 5, 8 and 10 had all told their parents about their pain and their parents had then suggested a course of action such as seeing a doctor (interviewees 3, and 10) or seeking treatments from providers such as a reiki healer and a physiotherapist (interviewees 5 and 8). Only interviewee 1 (an adult living independently) did not consult her family for specific back pain assistance, and raised the issue only in conversation with them.

Next, consider *incidental* search. Incidental search occurred when interviewees did not usually deliberately attempt to find information but were exposed to it from external sources such as from colleagues, friends, advertising signs on providers’ doors and talkback shows. For example, interviewees 6, 7, 11 and 12 were having a general conversation with a work colleague and the issue of back pain arose as a topic in the conversation. In brief, it became evident from the analysis that search effort is a function of deliberate or incidental search.

### Implications

The findings of this research have *implications* for public and private health sectors, and also for marketing management researchers. Persons with back pain rely mainly on internal information and external personal word of mouth by their doctor and friends which does not appear to provide an effective strategy for choosing treatment types and providers. Therefore, the public health system could provide more treatment related information about mainstream *and* complementary and alternative treatments. Furthermore they could provide generic information about how to select a service provider (for example, what steps to take to find a reputable and qualified service provider with the desired area of expertise) through a range of media. As a first option, information can be channeled through GP practices, in preference to the television and radio shows that have been shown to be more effective for the less information savvy, health conscious consumer [28]. However, research indicates that GPs may be unlikely to refer patients to complementary and alternative treatments due to their lack of belief in them [63]. This stance could be due to the fact that many GPs are not familiar with the research advances relating to alternative treatments like chiropractic care and massage. Therefore, an alternative solution is to use social workers who are trained in complementary and alternative treatments [62] and who understand physiology and anatomy. These professionals can assist patients to explore choices, discuss concerns, ask questions to increase understanding and make more informed decisions without judgment or bias.

As well, this information could be channeled through printed pamphlets/booklets in back health professional practices aimed at persons with back pain and their family and friends who are more health conscious and information savvy. Of course, the psychographic and media profiles of all these segments may need to be researched as a precursor to this action.

The findings of this study also have implications for health service providers. Knowledge of the information content required by interviewees can be used to better communicate services so that consumers can make better decisions. Service providers have two options: they can provide information about their service only and/or provide additional information about other competing treatment options available and compare the differences/benefits to their specific treatment offering. Service providers can also target this information through various media. Some of these media include more personal sources like the word of mouth of their own patients (who speak with friends and family) and office staff as well as their websites, pamphlets located in doctor’s practices (for more in-depth treatment and service related information), radio advertising, signage and the telephone directory (for contact details and other specific service provider related queries). In order to provide personal ratings of the practice and provider, the websites could host a patient review site where past and present patients can discuss the outcomes of their experiences and even suggest improvements. The website could also provide an appointment availability function whereby patients could book or cancel appointments and find out if appointment slots are available when needed.

Next, the research has implications for market researchers. The convergent interviewing methodology was an efficient and effective way of undertaking inductive research in an under-researched area and so could be used in similar research projects. However, two limitations are evident from this study, each providing grounds for *further research* into this topic. One limitation relates to the sample size, which although adequate for constructing a preliminary model of search behavior, requires statistical generalization (theory testing) through a survey to complement the analytic generalization (theory building) of this research [47]. A final limitation is that we were not able to find any strong deviations in interviewee’s answers and this could be because of the sample size. Further *quantitative* research about information search should be undertaken to investigate the presence of deviations in responses and investigate the roles of demographic, psychographic, behavioural and back pain characteristics on response differences.

## Conclusions

This study has explored how persons with back pain search for information when trying to choose from a range of treatment options and service providers. Three main issues were researched: where to search for information, what information to search for, and how much effort to expend searching. In terms of where to search for information, persons with back pain rely heavily on their own memories (internal) and external personal sources such as friends and doctors when searching for information about treatment types and service providers. However, more often than not, these perceptions are wrong, biased or too limited to provide a useful guide for decisions.

These findings differ from the findings of previous studies [30] about the types of information health consumers require when searching for information about alternative or mainstream healthcare services. First, internal search was not mentioned in the literature even though it is the source most used and appears to be inaccurate, and interviewees did not rely to any extent on pamphlets. The results have identified for the first time that limited information availability was only one of three categories of reasons identified about why persons with back pain do not search for more information particularly from external non-personal sources.

Next, this study confirms findings from other research [36] that information *content* sought by patients is health context specific. For example, some interviewees required information about self-help techniques for their back problem. Examples of service related information content included the speed with which an appointment with the provider could be obtained, a providers’ area of expertise and their reputation. In turn, this research outlined for the first time the exact information items sought by interviewees for both back pain treatment types and service providers, and its linkages to sources. Furthermore, extending previous studies [36], this research showed that the sheer number of treatment options and service providers required interviewees to undertake a more complex search step (combining internal search, and external personal and non-personal search) than in some other health care areas.

Finally, the information sources used by persons with back pain are a function of the information content they seek; this link is a new finding. For example, the use of external non-personal sources such as the telephone directory, contacting the provider directly or using practice signage was reserved for acquiring more detailed service provider information such as the speed of acquiring an appointment; whereas information about the provider’s reputation and expertise was obtained from internal and other external personal sources. This situation reflects the information available from each source rather than the information search preferences of these persons with back pain, as evidenced by the reasons given for their low reliance on external non-personal sources.

## Competing interests

The authors declare they have no competing interests.

## Authors’ contributions

HM conceived of the study, conceived the research design, conducted the interviews, undertook data analysis and wrote the first draft of the manuscript and approved the final manuscript. JG assisted in the research design, assisted in the data analysis and wrote additional sections of the second draft of the manuscript as well as writing the final version of the manuscript and approving the final manuscript. CP advised on research design and wrote some sections of the second draft of manuscript and approved the final manuscript.
